# Inclusion of Healthy Oils for Improving the Nutritional Characteristics of Dry-Fermented Deer Sausage

**DOI:** 10.3390/foods9101487

**Published:** 2020-10-18

**Authors:** Márcio Vargas-Ramella, Paulo E. S. Munekata, Mohammed Gagaoua, Daniel Franco, Paulo C. B. Campagnol, Mirian Pateiro, Andrea Carla da Silva Barretto, Rubén Domínguez, José M. Lorenzo

**Affiliations:** 1 Centro de Educação Superior da Região Sul—CERES da Universidade do Estado de Santa Catarina, Chapecó, Santa Catarina 89.800-000, Brazil; marcio.ramella@hotmail.com; 2 Centro Tecnológico de la Carne de Galicia, Rúa Galicia N° 4, Parque Tecnológico de Galicia, San Cibrao das Viñas, 32900 Ourense, Spain; paulosichetti@ceteca.net (P.E.S.M.); danielfranco@ceteca.net (D.F.); mirianpateiro@ceteca.net (M.P.); rubendominguez@ceteca.net (R.D.); 3 Food Quality and Sensory Science Department, Teagasc Ashtown Food Research Centre, Ashtown, Dublin 15, Ireland; gmber2001@yahoo.fr; 4 Departmento de Tecnologia e Ciência de Alimentos, Universidade Federal de Santa Maria, Santa Maria, Rio Grande do Sul CEP 97105-900, Brazil; paulocampagnol@gmail.com; 5 Department of Food Technology and Engineering, UNESP—São Paulo State University, Street Cristóvão Colombo, 2265, Sao Jose do Rio Preto 15054-000, Brazil; andrea.carla@unesp.br; 6 Área de Tecnología de los Alimentos, Facultad de Ciencias de Ourense, Universidad de Vigo, 32004 Ourense, Spain

**Keywords:** food reformulation, healthy meat product, game meat, fatty acids, volatile compounds, sensory properties

## Abstract

The influence of partial replacement of animal fat by healthy oils on composition, physicochemical, volatile, and sensory properties of dry-fermented deer sausage was evaluated. Four different batches were manufactured: the control was formulated with animal fat (18.2%), while in the reformulated batches the 50% of animal fat was substituted by olive, canola, and soy oil emulsions immobilized in Prosella gel. The reformulation resulted in a decrease of moisture and fat contents and an increase of protein and ash amount. Moreover, reformulated sausages were harder, darker, and had higher pH values. This fact is related to the lower moisture content in these samples. As expected, the fatty acid composition was changed by the reformulation. The use of soy and canola oils increased polyunsaturated fatty acids and omega-3 content and decreased n-6/n-3 ratio and saturated fatty acids. Thus, the use of these two oils presented the best nutritional benefits. The changes observed in the fatty acids reflected the fatty acid composition of the oils employed in the emulsions. Regarding volatile compounds (VOC), the replacement of animal fat by healthy emulsion gels increased the content of both total VOC and most of individual VOC. However, the lipid-derived VOC did not show this trend. Generally speaking, the control samples presented similar or higher VOC derived from lipid oxidation processes, which could be related to the natural antioxidant compounds present in the vegetable oils. Finally, all reformulated sausages presented higher consumer acceptability than control samples. In fact, the sausage reformulated with soy oil emulsion gel was the most preferred. Thus, as a general conclusion, the reformulation of deer sausages with soy emulsion gel improves both composition and sensory quality of the final product, which could be an excellent strategy to the elaboration of healthy fermented sausages.

## 1. Introduction

Over the last number of years, the consumption of game meat has increased. The characteristic texture and taste of the meat from game, such as wild deer is appreciated by the consumer [1]. Spain is the second world producer of deer venison which mainly exports meat from hunted animals [2]. According to official data, the capture of cynegetic deer in Spain surpassed 144,000 animals (red deer) in 2018. The average live weights of these animals were 80 kg and resulted in 11,530 tons with an economic value of 25 million euros. In addition to red deer, 66,737 roe deer and 24,337 fallow deer were hunted in the same year [3]. Between 2012 and 2018, the red deer captures ranged between 144,061 and 182,458 animals per year [3]. However, it is important to highlight that deer meat is simply considered as a by-product of hunting [4]. Additionally, the consumption of both fresh meat and traditional meat products elaborated with game meat has increased [1]. It is due to their particular taste and excellent nutritional characteristics (low fat and cholesterol contents and high amounts of other essential nutrients) [2,5,6,7,8]. Additionally, taking into account health aspects, it also has high amount of PUFA (39–50 g/100 g fatty acids) [8] and long-chain n-3 fatty acids [2,7,8,9].

A wide range of meat products can be produced with deer meat, including burgers [10] dry-cured cecina, pâté [9], dry-cured loin [11], and dry-fermented sausages such as chorizo [1,12,13] and salchichón [4,12,14,15]. However, the market for game products is very restricted [12] and these products are labelled as “gourmet products” in the international market. Nevertheless, due to the very lean character of meat of deer, the use of pork backfat or pork meat with high fat contents are necessary in the dry-fermented sausages elaboration in order to ensure the correct dry-ripening process [4,14]. This strategy is necessary because fat limits the mobility of the water from the interior of the meat product to the environment, which cause a progressive, slow, and controlled drying process. In fact, the use of an inadequate amount of fat (a very lean product) causes a rapid initial drying, which creates a superficial crust that prevents the correct subsequent drying process, affecting all the changes that occur during ripening and yielding a poor-quality product. Thus, with the addition of animal fat, the health benefits of the special composition of deer meat are masked by the high values of saturated fat and cholesterol of the pork backfat. In this regard, cynegetic venison sausages have around 40–50% of fat [4]. In fact, in an article that studied the proteolysis, free fatty acids, and composition of commercial dry-fermented deer sausages, the authors reported values of fat between 32% and 53% of dry matter (DM) in chorizo and between 33% and 42% of dry matter salchichón [12]. Similarly, other authors reported fat values of 31–36% (DM) in deer chorizo [1,13] and between 19% and 40% (DM) in salchichón [14,15]. Additionally, the saturated fatty acids accounted for more than 40% of total fatty acids in deer salchichón [14].

It is known that the diets rich in fat (particularly saturated fat) increase the risk of overweighting and developing chronic disorders, particularly ischemic heart disease, stroke, and cancer [16,17,18,19,20]. Furthermore, the consumers, aware that diet plays a crucial role in their health, have increased the demand for more healthy meat products [21]. Therefore, following the international recommendations and consumers’ demands, the meat industry has focused on two main strategies during the last decade.

A direct reduction of fat added in the sausages elaboration by using a higher proportion of lean meat is the first option. Several researchers studied this option [4,22,23,24,25]. However, the fat has a great influence on dry-fermented sausage quality. Sensory characteristics as appearance, tenderness, hardness, and overall palatability depend directly on the fat content [23,26]. The characteristic flavor of meat products, which is one of the most appreciated attributes for the consumers, are also related to fat. Additionally, fat is the precursor of the lipolysis and lipid oxidation reactions that modulate the release and formation of several volatile compounds, which are crucial in typical dry-cured aroma [27,28,29,30]. Thus, the numerous and complex reactions during ripening conferred the characteristic aroma of these products, and depend on the concentration and the olfactory threshold of each compound [31]. Moreover, as commented above, fat also exert a technological function during the dry-ripening process, because the granulated fat can facilitate the regular moisture release occurring during ripening phase [32]. Thus, the fat reduction strategy tended to result in lower sensory quality and technological problems.

With the aim to mitigate fat reduction drawbacks, multiple authors used fat replacers such as konjac gel [32], fructooligosaccharides [31,33,34], oat, and chia emulsion gel [35], cellulose gel [30], or boiled quinoa [20]. However, in most of these studies, the use of fat replacers only affected the total fat content, but did not affect or had low effect in the fatty acid profile. Therefore, in order to improve the nutritional value of fat, the most recent studies replaced animal fat by oil-in-water emulsions. In this regard, several researches indicated that the replacement of animal fat by different non-animal lipids (vegetable or marine oils) resulted in a reduction of both total fat and SFA contents [9,36,37,38,39], and also reduces the cholesterol content in the final product [37].

However, the correct choice of oils as substitutes for animal fat is the most important point of the reformulation strategy. The nutritional recommendations indicate that the intake of saturated fats should be replaced by monounsaturated or polyunsaturated fatty acids (mainly omega-3). Nevertheless, not all oils rich in these fatty acids are good candidates for the reformulation of meat products. Various authors have observed that the use of chia [9,10,38,40], linseed [9,10,40], or fish oils [36,41,42] (oils with high omega-3 content) in the reformulation of meat products have resulted in a significant increase in oxidation rates and/or rancid and fishy flavors, which decreased the consumer acceptability and sensory properties. For this reason, these oils have been ruled out for the design of this experiment. Following the recommendations, and taking into account the composition of multiple oils, the use of olive oil has been considered, since it contains a high amount of oleic acid (about 70% of total fatty acids) [43]. In fact, the use of olive oil as animal fat replacer in other meat products were carried out in previous studies with promising results [37,38]. Moreover, soybean oil presented also a favorable fatty acids profile, with high polyunsaturated fatty acids amounts (about 60%) and intermediate values of omega-3 fatty acids (5–11%) [44]. In addition to their composition, there are few studies that used soybean oil as fat replacers in meat products [44,45,46], which demonstrated its viability as animal fat replacer. Finally, canola oil presents high monounsaturated (mainly oleic acid; 60%) and polyunsaturated fatty acids amounts (36%), with intermediate α-linolenic acid contents (about 10%) [47]. This oil was previously used in the reformulation of chicken meatballs [47] and sausages [48], and the results demonstrated a significant nutritional improvement without affecting the sensory characteristics of the products (presented the same consumer acceptability as control samples). Based on these aspects, the use of olive, canola, and soybean oils was proposed in the present study.

On the one hand, in cooked meat products such as Frankfurt type sausages [38,41], pâté [9,42] or in fresh products such as burgers [39,40,49], the use of oil-in-water emulsion is easy. On the other hand, the application of emulsion gels in dry-fermented or dry-ripened products is more difficult. Drying and ripening stages are characterized by multiple and complex physicochemical changes and the emulsion gels must be stable throughout the process [50]. In order to overcome these issues, the use of emulsion gels (healthy oils immobilized in a gel structure) has been proposed in recent years [51]. In this sense, the application of these emulsions as animal fat replacer can confer the distinctive characteristics of saturated fat but with favorable fatty acid profile. Recent techniques of converting liquid healthy oils into a solid-like gel were explored by multiple researchers. Different dry-fermented sausages were reformulated with healthy oil-in-water emulsion gels using konjac [36,50,52,53], whey protein powder [54], carrageenan [55], or oleogels [56], which is a promising alternative to produce healthier fermented sausages [30].

However, as far as the authors are aware, the use of a healthy oil stabilized into an alginate-wheat glucose-phosphate matrix as a functional ingredient and animal fat replacement in the development of healthy dry-fermented sausage has not been explored. Thus, the effect of dry-ripening process in its viability and stability was not studied.

On the other hand, very few studies examined the effect of pork backfat replacement by healthy oils in deer dry-fermented sausages. To this regard, only two manuscripts were found about the influence of partial pork meat replacement by olive oil organogel in the sensory [15] or composition and nutritional values [14] of deer dry-fermented sausages. In these studies, the authors used pork meat with high fat content (around 50%) instead of pork backfat as we used in the present manuscript. These different strategies gave totally different results in both, the composition and the sensory quality.

With the aforementioned in mind, the objective of this research was to design a technological strategy for the incorporation of healthy oil-in-water gelled emulsions as a partial animal fat replacer to improve nutritional characteristics of dry-fermented deer sausages. The chemical and physicochemical characteristics, nutritional properties, volatile release modifications, sensory properties, and consumer acceptability were assessed.

## 2. Materials and Methods 

### 2.1. Preparation of Prosella Gel and Fatty Acid Characterization of Fat Sources

Elaboration of emulsion alginate-based hydrogels (Prosella gel) were carried out following the procedure described by Barros et al. [39]. Its preparation was a day before of sausages manufacture. Three different batches of emulsion hydrogels were obtained using olive oil, canola oil, and soy oil. The fatty acids composition of the fat sources used in the present study is shown in Table 1.

### 2.2. Manufacture of Dry-Fermented Sausages

For the present research, four different batches of fermented sausages were manufactured in the pilot plant of the Meat Technology Center of Galicia (San Cibrao das Viñas, Ourense, Spain). In control (CON) batch was used 100% of pork backfat as fat source. In the other ones, 50% of pork backfat was replaced by Prosella gel containing olive (OLI), canola (CAN), or soy (SOY) oils. An identical formula was used for all batches, except for fat source. The deer sausage formulation included lean deer meat (740 g·kg^−1^), fat source (182 g·kg^−1^), and a supplement “542 Salchichon” (46 g·kg^−1^) from Laboratorios Ceylamix (Valencia, Spain). No starter culture was added. All manufacture conditions were previously published by Bis-Souza et al. [31].

The manufacturing process was replicated with the same ingredients, formulation, and methods in two different months. Ten samples from each batch and elaboration were taken after 45 days of ripening.

### 2.3. Physicochemical, Lipid Oxidation, and Composition Analysis

The physicochemical (pH, color, and texture) and proximate composition was evaluated following the procedure described by Lorenzo et al. [36], while for lipid oxidation determination, the TBARS index was evaluated [57]. 

### 2.4. Fatty Acids Analysis

Total fat was extracted from 10 g of sample [39]. Then, the fatty acids were transesterified, and analyzed by gas chromatography-FID technique (Agilent Technologies, Santa Clara, CA, USA), following the conditions reported by Barros et al. [39]. The results were expressed as g/100 g of total identified fatty acids.

### 2.5. Volatile Compounds Analysis

The volatile compounds of 1 g of sample were analyzed using SPME-gas chromatography-mass spectrometry technique (Agilent Technologies, Santa Clara, CA, USA), following the procedure described by Domínguez et al. [28]. The volatile results were expressed as area units per gram of sample (AU × 10^4^/g of sample).

### 2.6. Sensory Analysis of Deer Sausages

The quantitative-descriptive analysis (QDA) was conducted with 15 trained panellists selected from the Meat Technology Centre of Galicia. The sausages were cut into slices (≈5 mm thick), coded with three numbers, and presented at room temperature. Nine sensory traits of deer sausages, grouped according to appearance (meat color and fat color), flavor (black pepper flavor, rancid flavor, and global flavor), odor, taste, and texture (hardness and chewiness) were assessed according to the methodology proposed by ISO regulations [58,59,60]. The intensity of every attribute was rated on a structured scale from 0 (sensation not perceived) to 9 (maximum of the sensation).

Sensory acceptability analysis was conducted by 68 consumers (29–40 years and from both genders) from Ourense (Spain) (Ethical Committee for Sensorial Analysis of Centro Tecnolóxico da Carne; approved number: SEN.CTC.20.001). The samples were evaluated to determine whether the panellist liked or disliked of the reformulated sausages in comparison to Control. The samples were three-digit coded and presented individually in unwrapped oblique slices approximately 5 mm thick on a small plate. The samples were randomly presented to consumers [61]. Consumers evaluated the deer sausages by the acceptance test using a 7-point hedonic scale (“1—disliked much”; “7—liked much”). Additionally, consumers also ordered the sausages according to their preference (structured 4-point scale: 1 = most favorite and 4 = less favorite).

### 2.7. Statistical Analysis

A total of 80 samples were used in the present study (10 sausages × 4 batches × 2 manufacture process replicates). Shapiro–Wilk analysis was used to test the normal distribution and variance homogeneity. Data from chemical composition, physicochemical parameters, fatty acids, and volatile compounds were examined using a one-way ANOVA analysis. Duncan’s test was used for the determination of differences between least squares means (*p* < 0.05), while correlations between variables were evaluated with the Pearson’s linear coefficient. All statistical analyses was achieved using IBM SPSS statistics for Windows (version 19.0. IBM Corp., New York, NY, USA).

The XLSTAT-Sensory version 2018 (Addinsoft SARL, Paris, France) software was utilized to determinate all the sensory data. For QDA analysis, a 2-way Mixed Model ANOVA was conducted (panellist and treatment). Dependent variables were intensity ratings corresponding to each sensory attribute. Duncans’ mean separation tests were used for post hoc analyses (*α* = 0.05). In addition, Friedman two-way ANOVA, was used to analyze preference data. Principal component analysis (PCA) was applied for the significantly different attributes and it was conducted to evaluate the deer sausages and, in this way, create the attribute maps.

## 3. Results and Discussion

### 3.1. Proximate Composition, Color, and Texture Parameters of Deer Sausages

The use of emulsion hydrogels (olive, canola, and soy oils) resulted in a significant decrease of moisture (*p* < 0.001) (Table 2). The CON samples presented a final moisture content of 39.79%, while in the experimental batches the moisture content varied between 27.69% and 29.90%. The moisture values found in the control samples were similar to those reported by other authors, who found moisture values about 34–45% in dry-fermented sausages [30,54]. In a similar way, the moisture values of experimental batches agree with those described by other authors (around 25–30%) also in dry-cured sausages [20,27,36,55,56].

Some authors also reported the lowest moisture amounts in reformulated dry-fermented sausages, in which animal fat was replaced by encapsulated oil-in-konjac matrix [36]. Additionally, the samples of dry-cured sausage reformulated with linseed oil oleogel (made with sterols) showed lower moisture content than the control samples [56]. In contrast, other researchers reported that the addition of oil-in-cellulose gel [30], beeswax oleogel [56], oil-in-konjac matrix [50], chia and oat emulsion gels [35], chia and olive oils structured in emulsion gels [62], and hazelnut oil pre-emulsified with whey protein [54] as animal fat replacers resulted in a significant increase of moisture content. It is well known that the drying-ripening process is vital for the progressive and adequate dehydration process of the product. Therefore, the differences observed between studies could be related to the different ripening conditions (time, temperature, relative humidity, air speed, etc.) as well as to the characteristics of the product (amount of fat, type of meat, casing size, etc.) or the behavior of the different fat substitutes (oleogels or emulsions, encapsulated oils, etc.).

The moisture differences found between CON and experimental batches suggest a faster drying process in sausages with animal fat replacement. It is well known that animal fat generates a barrier and reduces water loss during the drying step. In contrast, the oil-in-water emulsion gels used in the elaboration of OLI, CAN, and SOY samples contain 56% of water, which favors the drying process. Thus, our results showed that the barrier effect of Prosella gels is very low in comparison with animal fat. This aspect is an advantage for the manufacturer. In this sense, because the drying process is more intense and faster, the reformulated sausages can be produced in less time, with the economic and technological benefit that this result implies. Moreover, these variations in the final moisture between control and reformulated sausages will influence other parameters such as pH, texture, and sensorial characteristics.

Fat content (on a dry matter basis) showed a significant reduction with animal fat replacement. CON samples presented 37.62% of fat, while in the reformulated sausages were about 32.5%. Thus, the fat reduction between CON and reformulated samples were about 13–14%. Our values were similar to those reported for similar dry-fermented sausages [12,20,56]. As expected, the replacement of pork backfat, which contained about 80% of total fat [63], by oil-in-water emulsions that had only 37.2% of oil resulted in a significantly decrease of fat in the reformulated samples. These findings were reported by several authors, who proved that the use of gelled oils as animal fat replacers caused a total fat reduction in other similar dry-ripening sausages, such as sucuk [54], salchichón [36,56], fuet [62], and other types of fermented sausages [20,30,55].

In contrast, the animal fat replacement significantly enhanced the content of protein and ash. These results agree with those reported by other authors, who found an increase in protein and ash content when animal fat was replaced by boiled quinoa [20], amorphous cellulose gel [30], encapsulated oil in konjac matrix [36], or emulsion gel using protein isolate and gelatin [62]. These variations reflect the decrease in fat content, which results in a higher proportion of protein and ash in the dry matter of the reformulated sausages. Additionally, the variation in the ash content in the present study may be related to the addition of Prosella powder in the oil-in-water emulsion. This fact was also reported in a previous study in which beef burgers reformulated with oil emulsions using Prosella gel presented higher ash content than control samples [39]. Similarly, a study in which animal fat was replaced by chia and oat emulsion gels concluded that the concentration of minerals increased when emulsion gel was used in the fresh sausage formulation [35].

The reformulation also affected the pH of dry-fermented deer sausage. The samples with oil-in-water emulsions had higher pH values (*p* < 0.001) than CON sausages. The sausages from SOY batch presented the highest values (5.12; *p* < 0.05), followed by OLI and CAN (5.04) and finally the lowest values were observed in CON (4.48). Despite the pH changes, these pH values are consistent with those obtained in similar products [14,22,36,55,56]. Our pH level reached values less than 5.15 in all samples because of fermentation process and the acidification caused by lactic acid bacteria [30] and thus increased the microbial stability of the final product. The changes observed between the samples of different formulations could be related to the differences in moisture content among batches. In fact, as reported in a previous study [56], a negative and significant correlation was observed among pH and moisture values (*r* = −0.667; *p* < 0.01). Similarly, other authors also found that the animal fat replacement by emulsions gels resulted in higher pH values in the final product [35,36,62].

The instrumental color data obtained in this research was similar to those described by other authors in deer sausage [14]. As could be seen in Table 2, animal fat replacement by oil-in-water emulsions affected all color parameters. In this regard, the reformulated sausages were darker (*p* < 0.001) than CON samples. The highest L* values were observed in CON samples (40.81) followed by SOY (33.01) and finally OLI and CAN, with similar values (about 31). The same trend was observed by several authors in dry-fermented sausages reformulated with oil emulsions [20,24,36,62]. It is well-known that the fat amount has a strong influence on the L* values, since the higher the fat content in sausages, the higher the L* values [22]. Thus, the lightness reduction in reformulated sausages can be explained by the reduction of animal fat because this fat is white, and it provides the brilliant aspect of dry-fermented sausages [36]. Another possible explanation is that the higher dehydration process during ripening in the reformulated sausages resulted in lower L* values [24].

Regarding a* values, only SOY samples showed lower values (5.08; *p* < 0.05) than the other three batches (between 5.99 and 6.07). Despite these differences, it is worth mentioning that the values of all batches were similar. This means that the strategy of animal replacement by oil-in-water emulsions stabilized in Prosella gels gives rise to products that maintain the characteristic red color of the sausage.

As occurs with L* values, the yellowness (b*) also decreased as the animal fat was replaced by emulsion gels. In this sense, b* values of reformulated sausages were similar (between 4.81 and 5.17), while CON samples presented the highest values (8.49; *p* < 0.001). The same trend was observed by Lorenzo et al. [36], who found that the replacement of animal fat by encapsulated fish oil in konjac matrix produced a decreased in b* values. In contrast, other authors described that the use of emulsions or oleogels as fat replacers increased or did not affect the yellowness of the final product [54,56,62]. As reported by other authors, the differences between studies could be due to the characteristic color of the oleogelators or emulsifiers used in the production of the gels as well as the amount of oil used in the emulsion or the oleogel and their natural color. For example, the use of beeswax linseed oleogel in the reformulation of sausage resulted in higher b* values, and the authors attributed this fact to the yellow color of both oil and beeswax [56].

Texture parameters were also influenced by fat replacement. All parameters increased as the animal fat was substituted by oil-in-water emulsion gels. In accordance with our results, the formation of harder structures has been found as fat content decreased in the product [20,22,24]. Focusing on the hardness, which is the most important parameter from the consumer’s point of view, this parameter increased from 61.98 *N* in CON samples to about 275 *N* in OLI and CAN and 316 *N* in SOY samples. Similarly to our findings, the replacement of pork backfat by encapsulated fish oil in konjac matrix of pork sausage produced a progressive increase in hardness. Conversely, the use of beeswax oleogel or protein isolate/gelatin emulsion decreased the hardness of fuet [62]. Other authors also reported a decrease in the hardness values using oil-in-water emulsions in konjac matrix in different dry-fermented sausages [32,52]. It is well known that the dehydration process during ripening and the final moisture content is the most important parameter in the textural characteristics of this type of product. Thus, the differences observed between batches in the present research, as well as between the data of different studies could be attributed to the moisture content. In agreement with other studies about salchichón [22,36,56] and fuet [62], a significant and negative correlation between hardness and moisture content was observed (*r* = −0.904; *p* < 0.01). Similarly, springiness (*r* = −0.595; *p* < 0.01), cohesiveness (*r* = −0.614; *p* < 0.01), gumminess (*r* = −0.881; *p* < 0.01), and chewiness (*r* = −0.863; *p* < 0.01) also showed a significant and negative correlation with moisture. Thus, the present results corroborate that the moisture content is the most important factor that influences the texture parameters of the dry-fermented sausages.

### 3.2. Fatty Acids Composition of Deer Sausages

The reformulation of deer sausages significantly affected the content of fatty acids (Table 3). It is important to note that in all samples, monounsaturated fatty acids (MUFA) were the most abundant fatty acids, followed by saturated fatty acids (SFA) and polyunsaturated fatty acids (PUFA). Individually, in CON, OLI, and CAN samples the majority was the C18:1n-9, followed by C16:0, C18:2n-6, and C18:0. This profile was previously reported in similar dry-fermented sausages [14,36,55,56]. In contrast, in SOY samples the order was C18:1n-9 > C18:2n-6 > C16:0 > C18:0.

Regarding SFA content, the replacement of pork backfat by oil-in-water emulsions reduced SFA between 16.6% and 23.8%. Except for the contents of C20:0 and C22:0 (minor fatty acids; < 0.5% of total fatty acids), all individual SFA showed a significant reduction with the animal fat replacement. These findings were also observed by other authors, in which reformulated sausages with emulsified and gelled oils presented significantly lower SFA values than those formulated with pork fat [50,55,56,62]. 

The MUFA content varied among the batches. The samples from OLI and CAN had significantly higher values of total MUFA than CON, due mainly to the higher amounts of C18:1n-9 in these batches. However, the content of MUFA in SOY sausages was lower (*p* < 0.05) than CON samples. 

As occurs in MUFA, PUFA content also varied depending on the batch. In comparison with CON samples, total PUFA decreased in OLI samples and increased in CAN and SOY sausages. These differences are mainly due to the different contents of C18:2n-6 and to a lesser extent of C18:3n-3 between pork backfat and oils. In this regard, it is important to note that the improvement in the fatty acids profile of the reformulated samples are also related to the high content of C18:3n-3 when fat was replacement by oil-in-water emulsions. This increase was more pronounced in the CAN and SOY sausages. As a result of these variations, significant differences were also observed in the content of total n-3 and n-6 fatty acids. Thus, following the same trend described for C18:3n-3, the content of n-3 fatty acids increased in all reformulated batches. The highest concentration was observed in the SOY (2.83 g/100 g) batch followed by CAN (2.58 g/100 g), OLI (1.27 g/100 g), and finally CON (1.08 g/100 g). Similarly, the content of total n-6 was affected by the variation in the amount of C18:2n-6. In SOY (25.43 g/100 g) and CAN (16.26 g/100 g) samples the total n-6 content increased, while in the OLI (14.24 g/100 g) sausages n-6 value decreased in comparison with CON samples (15.79 g/100 g).

As commented above, several studies investigated the effect of the addition of oil-in-water emulsion as animal fat replacer in dry-fermented sausages [36,50,54,55,62]. Generally speaking, in all studies the authors found a significant reduction of SFA content and a progressive increased of MUFA and/or PUFA. However, the intensity of this effect depends on multiple factors, such as the level of animal fat replacement (partial or total replacement), the amount of oil used in the emulsions, and the type of oil used in the sausage manufacture. 

Thus, as a general conclusion for fatty acid composition, due to the low fat content of deer meat [7], dry-fermented sausages reflected the fatty acid composition of the fat or oil used in their manufacture. Therefore, the differences in fatty acids commented above are due to the fat/oil composition. The same trend was reported by other authors who observed that the fatty acid composition of the oil used in the sausage manufacture conditioned the fatty acids content of the final product [50,55,62].

Finally, due to the variations in total n-6 and n-3 fatty acids, the inclusion of emulsion gel in sausages significantly decreased of the n-6/n-3 ratio. This ratio is of great interest, because unbalanced diets with high n-6/n-3 ratio are associated with an increased risk of developing cardiovascular diseases, cancer and depressive disorders [64], while the consumption of n-3 has a protective effect against these diseases. Following nutritional recommendations, this ratio should be less than 4 [65], being the optimal value 1 [64]. In our study, all samples presented higher values than those recommended. The use of canola oil in the formulation of oil-in-water emulsion showed the best n-6/n-3 values (6.31), followed by SOY (9.00), OLI (11.20), and CON (14.66). Although these values were higher than recommended, the inclusion of emulsion oils resulted in a significant improve in the nutritional quality of the deer sausages.

### 3.3. Volatile Compounds of Deer Sausages

The reformulation significantly affected the levels of several volatiles organic compounds (VOC) of deer sausages (Table 4). After 45 days, a total of 84 compounds were identified in dry-fermented sausage samples. These compounds were grouped into seven chemical classes: terpenoids and benzene-derived compounds (34), aldehydes (16), alcohols (11), esters (8), ketones (6), acids (5), and others (4). 

Terpenoids and benzene-derived compounds were the predominant volatiles in all formulations, representing 71.3% of total VOC in CON samples and about 73% of total VOC in the reformulated sausages. In agreement with our findings, previous studies in salchichón showed that terpenes were the predominant class [22,27,28], representing between 58% and 71% of total VOC. The major terpene was *o*-cymene followed by 1R-α-pinene and β-phellandrene. Moreover, significant contents of thujene (both, α- and β-thujene), β-myrcene, α-phellandrene, 3-carene, D-limonene, terpinene (both, α- and δ-terpinene), safrole, and caryophyllene were also found in salchichón samples.

The same individual terpenes were previously reported by other authors in salchichón samples [22,27,28,31,66] and in other dry-cured sausages formulated with pepper [20]. A previous study found these terpenes in pepper [67,68]. However, some dry-cured products without added spices presented terpenes such as D-limonene, α- and β-pinene, or 3-carene, which could be related to the diet of the animals [68,69]. With these results it is easy to conclude that the VOC derived from spices clearly dominated the volatile profile of sausage, while the VOC derived from lipid oxidation, microbial metabolism, or other physicochemical changes contribute to a lesser extent to the total volatile compounds of this type of sausage. These terpenes have been described to contribute with fresh, menthol, herbal, and lemon notes [27,28,70].

Generally speaking, the reformulated sausages presented higher individual and total terpenoids and benzene-derive compounds (*p* < 0.001) (about 13,000 AU·10^4^/g) than CON samples (8821 AU·10^4^/g). Similarly, other authors reported that the reduced-fat sausages resulted in a higher terpenoid content [20,30]. This fact could be related to the fat that acts as solvent for these compounds, delaying their release [25]. Additionally, in the present study the content of terpenes among reformulated (OLI, CAN, and SOY) batches were similar (except some individual terpene). The higher VOC could be related to the low moisture content in the reformulated sausages. This fact is corroborated by the negative and significant correlation observed between pH and moisture values (*r* = −0.674; *p* < 0.001).

The second most important VOC in sausage samples were acids, representing about 15% of total VOC in all samples. Among them, acetic acid (1513 AU·10^4^/g in CON and about 2000 AU·10^4^/g in the other batches) was the major organic acid, followed by butanoic (341–683 AU·10^4^/g) and hexanoic acids (55.3–74.9 AU·10^4^/g). All these compounds were found in previous studies of salchichón [27,28,31,66]. Moreover, in agreement with our results, some authors reported that acetic acid was the most important organic acid in dry-fermented sausages [20,28,67]. The most probable origin of butanoic, pentanoic, and hexanoic acids in dry-fermented sausages is the carbohydrate fermentation induced by microorganisms, such as lactic bacteria and staphylococci [66,70]. However, the origin of butanoic acid, 3 methyl is the oxidation of its Streker aldehyde (butanal, 3 methyl). The acetic acid gives notes of ripened aroma, while butanoic acid gives cheese notes [27]. It is well known that the acids found in this research (butanoic, acetic, and butanoic, 3-methyl), together with 1-octen-3-ol have a vital role in the development of typical aroma of sausages [28,67]. As occurs in the terpenoids section, the reformulated sausages presented higher amounts of total and individual acids than CON samples. In this case, also a significant and negative correlation was observed between moisture and acids (*r* = −0.657; *p* < 0.001), which could explain the differences found in the VOC amounts.

The aldehydes content represents 4.8–5.4% of total VOC in reformulated samples and 6.6% in CON samples. In this group, the most abundant compound was the hexanal (except for OLI samples, in which the butanal, 3-methyl had the highest amounts). Other authors also found that hexanal was the most important aldehyde in dry-fermented sausages [22,27,30,36], while benzaldehyde or benzenacetaldehyde were found the most abundant aldehydes in other research [28,31]. In this study, other important aldehydes found in deer sausage samples were butanal, 2-methyl, butanal, 3-methyl, methylal, benzaldehyde, and benzeneacetaldehyde. The origin of the aldehydes could be grouped in two main routes. The linear aldehydes derived mainly from lipid oxidation [28,29], while the branched aldehydes are related to amino acid degradation and proteolysis [28,71]. The contents of pentanal, hexanal, or 2-octenal, depends on linoleic, linolenic, and arachidonic fatty acids, while the content of heptanal and nonanal derived from oleic fatty acid [29]. As could be seen in Table 4, the hexanal content was not influenced by the replacement of animal fat by emulsions containing canola or soy oils, while the samples reformulated with the olive oil presented a significantly lower value than the other batches. A similar trend was observed in the contents of pentanal, heptanal, and nonanal. Thus, OLI samples presented a significantly lower content of lipid-derived VOC than the other three batches (with similar values among them), which could be related to the high content of tocopherols in olive oil. It is well-known that the presence of tocopherols inhibit the lipid oxidation process [29], thus this fact could explain the lower values of these aldehydes in OLI samples. Moreover, it is also important to note that, besides the high unsaturated level of oils used in the present study, the VOC derived from lipid oxidation process did not have a significant increase in comparison with CON samples, which could also be related to the presence of natural antioxidants (phenolic compounds) in the vegetable oils. In contrast with our results, the reformulation of salchichón samples with encapsulated fish oil immobilized in konjac matrix resulted in a significant increase of lipid-derived aldehydes [36]. In another study, the use of emulsion gel with olive and chia oil mixture as animal fat replacer also increased the amounts of aldehydes in the fuet samples [62].

On the other hand, contrary to observed results for linear aldehydes, branched aldehydes and cycloaldehydes increased significantly as the animal fat was replaced by the emulsion gels. The origin of propanal, 2-methyl, butanal, 2-methyl and butanal, 3-methyl is the deamination-decarboxylation of the amino acids valine, isoleucine, and leucine, respectively [28,71]. The main route for the formation of benzaldehyde and benzeneacetaldehyde is the Streker degradation of some amino acids such as leucine or phenylalanine [72].

Regarding the aroma notes, linear aldehydes contributed with sweet, floral, grassy, and fruity notes. Hexanal presents a rancid aroma at high amounts, while in low content it gives a pleasant grassy aroma [28], and linear aldehydes derived from oleic acid oxidation had pleasant meaty notes [70]. The two cycloaldehydes detected in salchichón samples contributed with floral, acorn, and bitter almonds notes (benzaldehyde) and with acorn, rancid, and pungent aroma (benzeneacetaldehdye) [27,28]. Finally, propanal, 2-methyl had pungent and nutty odor and butanal, 3-methyl presented acorn-like, salty, fruity, and cheesy aroma [28]. Additionally, it is important to note that due to their low odor thresholds, aldehydes are one of the main VOC that contributed to the typical sausage aroma. Particularly, the content of butanal, 3-methyl was reported as important VOC that impart a characteristic “ripened flavor” [28,70].

Alcohols represented between 2.35% and 3.94% of total VOC. The major alcohol was the 2,3-butanediol, followed by glycidol, linalool, 1-octen-3-ol, and 1-hexanol. These compounds and 1-pentanol or 1-octanol are commonly detected in fermented sausage samples [20,27,28,31]. The alcohols of fermented sausages are mainly generated from the reduction of aldehydes [66]. As commented above, the 1-octen-3-ol is described as an important VOC that contributed to characteristic aroma of dry-cured products [28,71]. This compound is derived from the oxidation process of linoleic acid [28,29]. Similarly, 1-pentanol is derived from the degradation of hydroperoxides and 1-hexanol from reduction of hexanal, while the 1-octanol arises from oleic acid oxidation [28,29,67]. As occurs in other VOC, the content of total alcohols was higher in reformulated than in CON sausages, mainly due to the higher amounts of 2,3-butanediol, glycidol, and linalool in these samples. A similar trend was described in another study in which the authors found 2,3-butanediol only in fat reduced sausages [20]. In contrast, the content of lipid-derived alcohols did not show a clear trend, and CON samples presented the highest values of some of them such as 1-hexanol of 1-pentanol. This fact is in accordance with the content of linear aldehydes, in which OLI samples presented the lowest values of lipid oxidation VOC. Despite this result, a significant and negative correlation was observed between moisture and total alcohols (*r* = −0.855; *p* < 0.001), which could explain the differences found in the VOC amounts.

The content of ketones and esters were similar (about 1.5% of total VOC). The most abundant ketone was the acetoin, followed by 2-butanone, butyrolactone, 3-pentanone, and 2,3-butanedione. Other authors also reported in salchichón that acetoin was the major ketone [28,31]. The origin of linear ketones is lipid oxidation of free fatty acids, while acetoin are formed through Maillard reactions [28]. Some compounds of this class are 2-butanone and 2-heptanone (give a characteristic blue cheese aroma and has an intense odor) and the acetoin (buttery and sweet odor and a very low odor threshold) [28]. However, high amounts of butyrolactone in sausage were previously reported [28]. This VOC contributes with pleasant butter, fatty, creamy, fruity, and coconut-like nuances. Regarding esters, butanoic acid, ethyl ester was the most abundant, followed by the propanoic acid, ethyl ester. The main origin of esters in meat product is the esterification of carboxylic acids and alcohols, while the low molecular weight esters can be also a product of carbohydrate metabolism [28]. In the present study, seven out of eight detected esters were ethyl esters. It is well-known that ethyl esters have lower odor thresholds than methyl esters, thus they have more impact on the overall aroma of the product. Additionally, ethyl esters contributed to a proper fermented sausage odor and mask rancid notes [70]. In both cases (ketones and esters), the total amount was higher in reformulated samples than in control samples.

Finally, due to all-mentioned differences between samples, the total VOC compounds were significantly higher (17,854–19,027 AU·10^4^/g) in reformulated samples than in CON samples (12,378 AU·10^4^/g). Additionally, a negative and significant correlation between moisture and total VOC was also observed (*r* = −0.723; *p* < 0.001). With this in mind, it could be concluded that the higher VOC in reformulated samples are related to the lower moisture content in these samples, which determined that the volatile compounds are proportionally in greater amount in the reformulated batches than in the control batch. Conversely, the lipid-derived VOC did not show this trend, and, in some cases, the CON samples presented significantly higher VOC content of that derived from lipid oxidation processes, which could be related to the natural antioxidant compounds present in the vegetable oils.

### 3.4. Sensory Analysis of Deer Sausages

**Figure 1:**
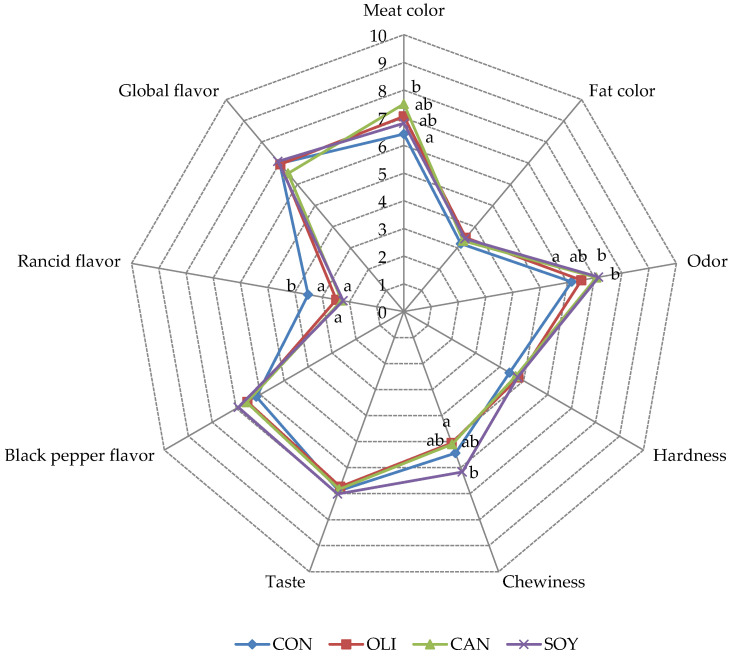
Effect of pork fat replacement by vegetable oil emulsion on sensory characteristics of dry-fermented deer sausages. ^a,b^ Mean values in the same row (corresponding to the same parameter) not followed by a common letter differ significantly (*p* < 0.05; Duncan test); Treatments: CON: sausages prepared 100% pork fat; OLI: sausages reformulated with 50% of pork fat replaced by olive oil; CAN: sausages reformulated with 50% of pork fat replaced by canola oil; SOY: sausages reformulated with 50% of pork fat replaced by soy oil.

The results of the descriptive sensorial analysis for the evaluated attributes in deer sausage are shown in Figure 1. From all attributes, only meat color, odor, chewiness, and rancid flavor were influenced by the reformulation. To this regard, the samples from CON batch presented the lowest scores for meat color attribute, which could be explained by lower L* values (darker) of the reformulated samples, as previously discussed in the color parameters section.

The “odor” was significantly higher in the reformulated sausages (mainly CAN and SOY samples) than in the CON group. This fact could be explained by the lower values of total VOC found in CON samples in comparison with the values found in reformulated samples. Similarly, the sensory analysis revealed significantly higher “rancid flavor” in CON than in reformulated sausages, which could be related to the highest amounts of lipid-derived volatiles in these samples. As discussed in the volatile section, in most cases, the content of volatiles from lipid oxidation was higher in the control samples, which is clearly reflected in the scores of the sensory panel. As occurs in our research, Alejandre et al. [55] also found differences in odor between control and sausages formulated with linseed emulsion gel as animal fat replacer. Moreover, another study also reported that the replacement of animal fat by amorphous cellulose gel in dry-fermented sausages resulted in positive scores for aromatic attributes than control samples [30]. 

On the other hand, although panelists found higher “black pepper flavor” in reformulated samples than in CON, these differences were not significant. These results are also supported by the volatile analysis, due to presented higher amounts of terpenoids and benzene-derived compounds in reformulated sausages, which are mainly derived from spices.

It is important to note that panelists described higher scores of “hardness” in reformulated samples than in CON sausages (*p* > 0.05). This fact is related to the clear differences found in the texture analysis, and due to the lowest value of moisture in reformulated sausages. Contrary to our results, a previous study in dry-fermented sausage found that the reformulation process with oil-in-water emulsion immobilized in konjac gel decreased all sensorial parameters [50]. These authors found that the use of oil-in-konjac matrix resulted in a softer structure in meat product, decreasing the firmness scores, which justified the lower sensorial scores in the reformulated sausages. Similarly, other authors also observed that fuet (a fermented sausage similar to salchichón) reformulated with oleogel or emulsion gel presented lower scores for all attributes than samples formulated with animal fat [62].

**Figure 2:**
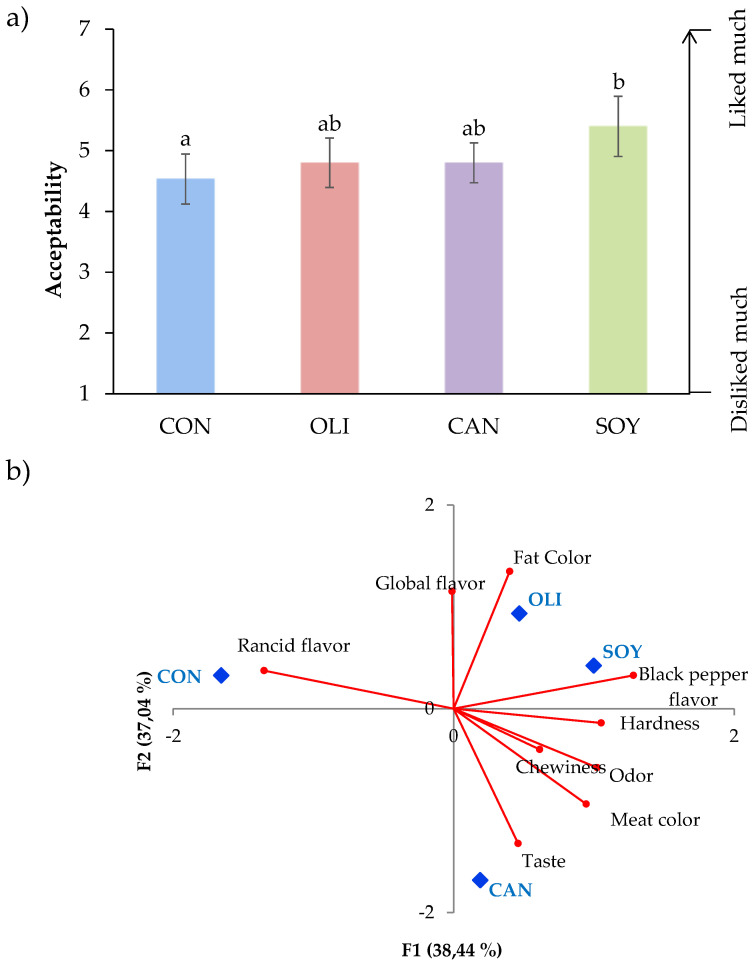
Global acceptance of dry-fermented deer sausages reformulated with healthy oils (**A**) and projection of the sensory attributes and sample batch in the plane defined by the first two components (**B**). ^a–b^ The bars of the Figure 2A with different letter differ significantly (*p* < 0.05; Duncan test).

Regarding acceptance test, all batches obtained a score higher than four (acceptability limit; Figure 2A). Sausages from CON batch presented the lowest sores (4.53), while reformulated samples with olive and canola oils had intermediate values (4.8 both) and SOY samples the highest values (5.40) (significantly higher than control). Although the results show higher acceptability scores in all reformulated samples, it should be noted that the acceptability of the sausages with olive oil and canola did not show significant differences with the control samples, while the reformulated sausages with soy oil obtained significantly higher values than the control samples. With these results, it could be concluded that the reformulation of deer sausage using oil-in-water emulsions immobilized with Prosella gel does not affect or increase acceptance of the final product. Additionally, there are no significant differences among reformulated samples. Our findings agree with the results reported in another study with deer fermented sausage, who found that the replacement of up to 25% of pork meat (with 50% of fat) by olive oil immobilized in a protein concentrate organogel resulted in a similar appearance and odor to the control samples [14]. Other authors also found that the inclusion of amorphous cellulose gel up to 50% did not influence the sensory properties of fermented sausages. In contrast, the use of oil-in-konjac matrix in chorizo [50], emulsion or oleogel in fuet [62], and oleogels as replacer of 40% of fat in salchichón [56] resulted in a significant decrease in consumers acceptance.

An acceptable differentiation between batches was possible with PCA (Figure 2B). The attribute map of the sausages showed 75.48% of total variability (F1 and F2 explained 38.44% and 37.04% of total variability, respectively). The attributes more influenced on F1 were fat color, taste and global flavor, while black pepper flavor, rancid flavor, hardness, odor, and meat color had higher weight in F2.

The spatial separation showed that batches were separated in two different groups. One of them represented by CON samples and the second one by sausages from OLI, SOY, and CAN batches. 

**Figure 3:**
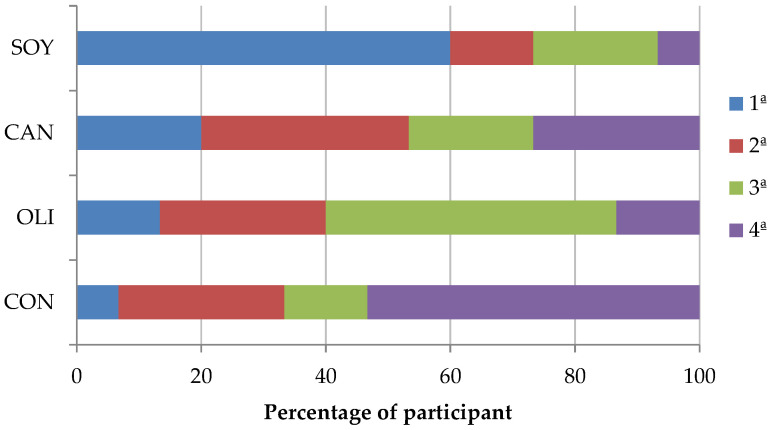
Preference attributed by the panelist on dry-fermented deer sausages.

Finally, regarding preference ordination of the different treatments, there were significant differences (Figure 3). Freedman test showed that less than 8% of consumers would choose CON as the most preferred. In contrast, SOY sausages obtained the highest scores, being chosen by 60% of consumers. Intermediate values were obtained by OLI and CAN samples, chosen by 13% and 20% of consumers, respectively.

The sensory characteristics indicated that the use of soy oil emulsion immobilized in Prosella gel resulted in higher consumer acceptability of the final product than control samples. Thus, vegetable oil emulsion immobilized in Prosella gel could be used as a suitable alternative of animal fat in the reformulation of fermented sausages.

## 4. Conclusions

The use of vegetable oils structured into emulsion gel shows a strong potential for application in the meat industry, especially for the development of healthier dry-fermented sausages. The strategy of replacing animal fat by oils gives rise products with good color and texture characteristics, improves the chemical composition and fatty acids profile, increases the odor characteristics of the final product, and inhibits the lipid oxidation process. Additionally, the sensory results indicated that reformulated sausages presented higher acceptability than traditional samples and the use of soy oil is the most preferred reformulation strategy for the consumers. It is important to note that, from technological point of view, the use of vegetable oil emulsions immobilized in Prosella gel resulted in faster drying process, which could be economically interesting for the manufacturers. Thus, as a general conclusion, the use of vegetable oils emulsions in Prosella gel as animal fat replacer in dry-fermented sausages is technologically viable and improves the composition and nutritional quality, oxidation stability, and sensory properties of the final product.

## Figures and Tables

**Table 1:** Fatty acid composition (expressed as g/100 g of total fatty acids) of fat sources.

	Fat Source			
	Pork Backfat	Olive Oil	Canola Oil	Soy Oil
C14:0	1.19 ± 0.002	0.03 ± 0.003	0.06 ± 0.005	0.08 ± 0.002
C16:0	23.03 ± 0.16	11.73 ± 0.04	4.96 ± 0.07	10.50 ± 0.02
C16:1n-7	1.89 ± 0.07	0.84 ± 0.11	0.18 ± 0.01	0.09 ± 0.003
C17:0	0.23 ± 0.001	0.08 ± 0.000	0.05 ± 0.006	0.09 ± 0.001
C17:1n-7	0.19 ± 0.007	0.12 ± 0.003	0.06 ± 0.002	0.05 ± 0.001
C18:0	12.74 ± 0.22	3.40 ± 0.02	2.01 ± 0.05	3.31 ± 0.008
C18:1n-9	41.18 ± 0.04	72.16 ± 0.06	54.65 ± 0.002	24.99 ± 0.006
C18:1n-7	2.67 ± 0.005	2.21 ± 0.04	2.68 ± 0.11	1.55 ± 0.02
C18:2n-6	13.68 ± 0.23	7.70 ± 0.022	27.44 ± 0.05	52.10 ± 0.05
C20:0	0.24 ± 0.000	0.41 ± 0.004	0.48 ± 0.001	0.33 ± 0.001
C18:3n-6	0.04 ± 0.000	0.03 ± 0.001	0.33 ± 0.001	0.26 ± 0.001
C20:1n-9	0.87 ± 0.03	0.24 ± 0.003	0.97 ± 0.02	0.22 ± 0.001
C18:3n-3	0.66 ± 0.01	0.61 ± 0.000	5.30 ± 0.01	5.70 ± 0.02
C20:2n-6	0.64 ± 0.02	n.d.	0.05 ± 0.001	0.04 ± 0.002
C22:0	0.01 ± 0.001	0.14 ± 0.001	0.39 ± 0.000	0.47 ± 0.002
C20:3n-3	0.11 ± 0.004	0.24 ± 0.000	n.d.	n.d.
C20:4n-6	0.23 ± 0.04	n.d.	n.d.	n.d.
C24:0	n.d.	0.06 ± 0.002	0.15 ± 0.001	0.17 ± 0.003
SFA	37.61 ± 0.38	15.85 ± 0.07	8.14 ± 0.13	14.99 ± 0.03
MUFA	45.93 ± 0.05	75.33 ± 0.09	57.74 ± 0.10	26.69 ± 0.03
PUFA	15.59 ± 0.29	8.58 ± 0.02	33.15 ± 0.05	58.10 ± 0.06
n-6	14.75 ± 0.29	7.73 ± 0.02	27.84 ± 0.06	52.40 ± 0.05
n-3	0.77 ± 0.005	0.85 ± 0.001	5.30 ± 0.001	5.70 ± 0.02

SFA: saturated fatty acids; MUFA: monounsaturated fatty acids; PUFA: polyunsaturated fatty acids; n.d.: not detected.

**Table 2:** Effect of fat source on physicochemical and composition parameters of dry-fermented deer sausage.

	Fat Source				Sig.
	CON	OLI	CAN	SOY	
**Composition (g/100 g)**					
Moisture	39.79 ± 0.72 ^c^	29.68 ± 0.41 ^b^	29.90 ± 0.43 ^b^	27.69 ± 0.38 ^a^	***
Fat (dry matter)	37.62 ± 0.52 ^b^	32.53 ± 0.56 ^a^	32.30 ± 0.35 ^a^	32.54 ± 0.42 ^a^	***
Protein (dry matter)	47.39 ± 0.44 ^a^	49.60 ± 0.32 ^b^	49.53 ± 0.32 ^b^	49.43 ± 0.29 ^b^	***
Ashes (dry matter)	7.24 ± 0.07 ^a^	8.77 ± 0.21 ^b^	8.63 ± 0.06 ^b^	8.59 ± 0.06 ^b^	***
pH	4.84 ± 0.02 ^a^	5.04 ± 0.01 ^b^	5.04 ± 0.03 ^b^	5.12 ± 0.02 ^c^	***
**Color Parameters**					
L*	40.81 ± 0.63 ^c^	30.77 ± 0.40 ^a^	30.97 ± 0.46 ^a^	33.01 ± 0.59 ^b^	***
a*	5.99 ± 0.19 ^b^	6.02 ± 0.27 ^b^	6.07 ± 0.32 ^b^	5.08 ± 0.20 ^a^	*
b*	8.49 ± 0.25 ^b^	5.17 ± 0.31 ^a^	4.81 ± 0.23 ^a^	5.03 ± 0.26 ^a^	***
**Texture Parameters**					
Hardness (N)	61.98 ± 3.10 ^a^	279.47 ± 6.91 ^b^	271.56 ± 6.37 ^b^	316.18 ± 6.57 ^c^	***
Springiness (mm)	0.45 ± 0.01 ^a^	0.54 ± 0.005 ^b,c^	0.54 ± 0.01 ^c^	0.52 ± 0.005 ^b^	***
Cohesiveness	0.29 ± 0.01 ^a^	0.34 ± 0.005 ^b^	0.34 ± 0.003 ^b^	0.35 ± 0.003 ^b^	***
Gumminess (N)	18.36 ± 1.05 ^a^	96.07 ± 3.23 ^b^	92.83 ± 2.38 ^b^	109.54 ± 2.25 ^c^	***
Chewiness (N·mm)	8.40 ± 0.62 ^a^	51.44 ± 1.81 ^b^	50.24 ± 1.37 ^b^	56.49 ± 1.35 ^c^	***

^a–d^ Mean values in the same row (corresponding to the same parameter) not followed by a common letter differ significantly (*p* < 0.05; Duncan test); Sig.: significance: * (*p* < 0.05), *** (*p* < 0.001); SEM: standard error of the mean. Treatments: CON: sausages prepared 100% pork fat; OLI: sausages reformulated with 50% of pork fat replaced by olive oil; CAN: sausages reformulated with 50% of pork fat replaced by canola oil; SOY: sausages reformulated with 50% of pork fat replaced by soy oil.

**Table 3:** Effect of fat source on fatty acid composition (expressed as g/100 g of total fatty acids) of dry-fermented deer sausage.

	Fat Source				Sig.
	CON	OLI	CAN	SOY	
C14:0	1.32 ± 0.01 ^d^	1.02 ± 0.01 ^b^	1.00 ± 0.01 ^a^	1.08 ± 0.01 ^c^	***
C15:0	0.09 ± 0.002 ^b^	0.08 ± 0.001 ^a^	0.08 ± 0.001 ^a^	0.10 ± 0.002 ^b^	***
C16:0	22.68 ± 0.05 ^d^	19.10 ± 0.08 ^c^	17.28 ± 0.06 ^a^	18.67 ± 0.07 ^b^	***
C16:1n-7	2.12 ± 0.01 ^d^	1.75 ± 0.01 ^c^	1.65 ± 0.01 ^b^	1.62 ± 0.01 ^a^	***
C17:0	0.41 ± 0.002 ^c^	0.33 ± 0.002 ^a^	0.34 ± 0.002 ^a^	0.36 ± 0.003 ^b^	***
C17:1n-7	0.30 ± 0.003 ^c^	0.25 ± 0.002 ^a^	0.26 ± 0.001 ^b^	0.26 ± 0.002 ^b^	***
C18:0	12.12 ± 0.05 ^d^	9.79 ± 0.05 ^b^	8.92 ± 0.04 ^a^	10.02 ± 0.05 ^c^	***
9t-C18:1	0.29 ± 0.01 ^c^	0.26 ± 0.01 ^b^	0.22 ± 0.01 ^a^	0.26 ± 0.01 ^b^	***
C18:1n-9	39.69 ± 0.07 ^b^	48.14 ± 0.12 ^d^	46.89 ± 0.13 ^c^	35.40 ± 0.12 ^a^	***
C18:1n-7	2.58 ± 0.02 ^b^	2.41 ± 0.02 ^a^	2.74 ± 0.01 ^c^	2.36 ± 0.03 ^a^	***
C18:2n-6	14.29 ± 0.09 ^b^	12.90 ± 0.09 ^a^	14.90 ± 0.11 ^c^	24.11 ± 0.19 ^d^	***
C20:0	0.22 ± 0.001 ^a^	0.27 ± 0.001 ^b^	0.31 ± 0.002 ^d^	0.29 ± 0.001 ^c^	***
C18:3n-6	0.05 ± 0.001 ^a^	0.06 ± 0.001 ^a^	0.14 ± 0.002 ^c^	0.13 ± 0.001 ^b^	***
C20:1n-9	0.93 ± 0.003 ^c^	0.73 ± 0.003 ^a^	1.02 ± 0.003 ^d^	0.77 ± 0.003 ^b^	***
C18:3n-3	0.67 ± 0.01 ^a^	0.87 ± 0.01 ^b^	2.20 ± 0.04 ^c^	2.43 ± 0.03 ^d^	***
C20:2n-6	0.62 ± 0.004 ^d^	0.45 ± 0.003 ^c^	0.40 ± 0.004 ^b^	0.36 ± 0.002 ^a^	***
C22:0	0.06 ± 0.001 ^a^	0.09 ± 0.001 ^b^	0.15 ± 0.001 ^c^	0.21 ± 0.002 ^d^	***
C20:3n-6	0.13 ± 0.002 ^c^	0.12 ± 0.002 ^b^	0.11 ± 0.002 ^a^	0.11 ± 0.001 ^a^	***
C20:3n-3	0.09 ± 0.001 ^c^	0.07 ± 0.001 ^b^	0.06 ± 0.001 ^a^	0.06 ± 0.001 ^a^	***
C20:4n-6	0.58 ± 0.01 ^a^	0.63 ± 0.02 ^b^	0.61 ± 0.01 ^a,b^	0.63 ± 0.01 ^b^	*
C20:5n-3	0.09 ± 0.004	0.09 ± 0.004	0.09 ± 0.01	0.10 ± 0.004	ns
C22:5n-6	0.12 ± 0.002 ^b^	0.10 ± 0.002 ^a^	0.09 ± 0.002 ^a^	0.09 ± 0.001 ^a^	***
C22:5n-3	0.18 ± 0.004 ^a^	0.20 ± 0.01 ^b^	0.20 ± 0.005 ^b^	0.20 ± 0.003 ^b^	**
C22:6n-3	0.04 ± 0.001 ^a,b^	0.04 ± 0.001 ^a,b^	0.04 ± 0.001 ^a^	0.04 ± 0.000 ^b^	*
SFA	37.08 ± 0.09 ^c^	30.81 ± 0.14 ^b^	28.23 ± 0.10 ^a^	30.91 ± 0.12 ^b^	***
MUFA	45.68 ± 0.08 ^b^	53.34 ± 0.11 ^d^	52.65 ± 0.13 ^c^	40.49 ± 0.14 ^a^	***
PUFA	16.94 ± 0.12 ^b^	15.58 ± 0.13 ^a^	18.91 ± 0.17 ^c^	28.34 ± 0.22 ^d^	***
n-6	15.79 ± 0.11 ^b^	14.24 ± 0.11 ^a^	16.26 ± 0.13 ^c^	25.43 ± 0.19 ^d^	***
n-3	1.08 ± 0.01 ^a^	1.27 ± 0.02 ^b^	2.58 ± 0.04 ^c^	2.83 ± 0.03 ^d^	***
n-6/n-3	14.66 ± 0.07 ^d^	11.20 ± 0.08 ^c^	6.31 ± 0.05 ^a^	9.00 ± 0.04 ^b^	***

^a–d^ Mean values in the same row (corresponding to the same parameter) not followed by a common letter differ significantly (*p* < 0.05; Duncan test); Sig.: significance: * (*p* < 0.05), ** (*p* < 0.01), *** (*p* < 0.001), ns (not significant); SEM: standard error of the mean; SFA: saturated fatty acids; MUFA: monounsaturated fatty acids; PUFA: polyunsaturated fatty acids. Treatments: CON: sausages prepared 100% pork fat; OLI: sausages reformulated with 50% of pork fat replaced by olive oil; CAN: sausages reformulated with 50% of pork fat replaced by canola oil; SOY: sausages reformulated with 50% of pork fat replaced by soy oil.

**Table 4:** Effect of fat source on volatile compounds (expressed as AU·10^4^/g) of dry-fermented deer sausage.

Compound Information				Fat Source			Sig.
Name	LRI	m/z	Control	Olive Oil	Canola Oil	Soy Oil	
Glycidol	468	44	28.76 ± 1.33 ^a^	70.80 ± 3.51 ^c^	60.79 ± 2.83 ^b^	65.44 ± 1.67 ^b,c^	***
1-Propanol	546	59	9.19 ± 1.10 ^d^	5.29 ± 0.50 ^b^	7.25 ± 0.34 ^c^	3.21 ± 0.16 ^a^	***
2-Butanol, (R)-	583	45	5.13 ± 0.47	4.48 ± 0.43	5.54 ± 0.41	5.25 ± 0.35	ns
1-Butanol	696	56	6.59 ± 0.49 ^b^	4.66 ± 0.37 ^a^	5.26 ± 0.54 ^a^	4.05 ± 0.25 ^a^	***
(R)-(-)-2-Pentanol	744	45	3.04 ± 0.18 ^b^	1.56 ± 0.11 ^a^	1.38 ± 0.08 ^a^	7.04 ± 0.37 ^c^	***
1-Pentanol	851	70	18.45 ± 1.26 ^c^	9.67 ± 0.36 ^a^	13.75 ± 1.05 ^b^	14.67 ± 0.73 ^b^	***
2,3-Butanediol	922	45	119.2 ± 9.13 ^a^	412.7 ± 16.80 ^c^	327.6 ± 16.69 ^b^	489.4 ± 24.39 ^d^	***
1-Hexanol	971	56	32.61 ± 2.01 ^c^	14.80 ± 0.72 ^a^	23.00 ± 2.30 ^b^	21.07 ± 0.93 ^b^	***
1-Octen-3-ol	1080	57	35.24 ± 1.83 ^b^	26.94 ± 0.91 ^a^	36.76 ± 1.14 ^b,c^	39.84 ± 1.18 ^c^	***
1-Octanol	1165	84	7.11 ± 0.34 ^a^	12.44 ± 1.34 ^b^	11.61 ± 0.36 ^b^	10.92 ± 0.86 ^b^	***
Linalool	1185	71	26.64 ± 0.92 ^a^	44.08 ± 2.32 ^b^	49.90 ± 1.73 ^c^	43.37 ± 2.35 ^b^	***
**Total Alcohols**			292.0 ± 11.09 ^a^	607.4 ± 15.72 ^c^	542.8 ± 17.04 ^b^	704.2 ± 22.78 ^d^	***
Propanal	496	58	45.25 ± 3.23 ^b^	87.61 ± 3.25 ^c^	10.88 ± 1.24 ^a^	6.94 ± 0.40 ^a^	***
Propanal, 2-methyl-	529	72	10.01 ± 0.72 ^a^	32.23 ± 1.10 ^c^	29.77 ± 1.37 ^b,c^	27.70 ± 0.74 ^b^	***
Butanal, 3-methyl-	640	58	46.75 ± 1.96 ^a^	163.04 ± 3.68 ^c^	149.72 ± 7.49 ^c^	135.07 ± 4.91 ^b^	***
Butanal, 2-methyl-	654	57	23.55 ± 1.20 ^a^	83.42 ± 2.64 ^c^	74.17 ± 2.90 ^b^	69.61 ± 3.39 ^b^	***
Pentanal	717	58	35.68 ± 3.17 ^b^	26.32 ± 1.06 ^a^	34.51 ± 1.33 ^b^	44.89 ± 2.26 ^c^	***
2-Butenal, 2-methyl-	801	84	3.08 ± 0.19 ^a^	4.83 ± 0.26 ^b^	7.11 ± 0.45 ^c^	4.39 ± 0.28 ^b^	***
Hexanal	872	56	249.3 ± 27.99 ^b^	83.76 ± 4.08 ^a^	209.8 ± 18.21 ^b^	209.9 ± 13.97 ^b^	***
Methylal	903	45	192.2 ± 13.73	157.1 ± 9.73	183.9 ± 7.64	174.9 ± 6.28	ns
Heptanal	993	70	13.85 ± 1.53 ^c^	7.82 ± 0.39 ^a^	11.69 ± 0.86 ^b,c^	9.96 ± 0.66 ^a,b^	***
Hexanal, 5-methyl-	993	55	9.11 ± 1.44 ^c^	3.83 ± 0.19 ^a^	6.28 ± 0.40 ^b^	4.66 ± 0.26 ^a,b^	***
Methional	1022	48	13.31 ± 0.87 ^a^	32.75 ± 0.97 ^b^	35.97 ± 1.26 ^c^	31.29 ± 0.59 ^b^	***
Benzaldehyde	1072	106	99.77 ± 7.53 ^a^	125.2 ± 2.69 ^b^	152.8 ± 12.04 ^c^	111.6 ± 4.72 ^a,b^	***
Benzeneacetaldehyde	1155	91	57.85 ± 3.38 ^a^	58.51 ± 3.43 ^a^	89.15 ± 3.89 ^b^	85.82 ± 5.59 ^b^	***
2-Octenal, (E)-	1160	55	12.98 ± 3.53	11.17 ± 0.41	13.49 ± 1.06	11.11 ± 0.62	ns
Nonanal	1187	98	7.94 ± 0.61 ^c^	5.84 ± 0.24 ^a^	7.45 ± 0.49 ^b,c^	6.32 ± 0.28 ^a,b^	**
3-Isopropylbenzaldehyde	1321	148	2.95 ± 0.20 ^a^	3.55 ± 0.15 ^b^	3.66 ± 0.18 ^b^	3.37 ± 0.20 ^a,b^	*
**Total Aldehydes**			823.5 ± 27.86 ^a^	887.0 ± 19.57 ^a,b^	1020 ± 26.97 ^c^	937.6 ± 23.93 ^b^	***
Carbon disulfide	504	76	18.95 ± 2.78 ^b^	19.23 ± 2.08 ^b^	12.76 ± 0.88 ^a^	13.19 ± 1.31 ^a^	*
Furan, 2-pentyl-	1065	81	14.09 ± 0.92 ^a,b^	11.85 ± 0.64 ^a^	15.77 ± 1.07 ^b^	15.79 ± 0.55 ^b^	**
1,3-Benzenediol, monobenzoate	1072	77	39.70 ± 1.48 ^a^	56.20 ± 1.71 ^b^	67.41 ± 2.62 ^c^	54.23 ± 1.83 ^b^	***
Hexane, 2,4,4-trimethyl-	1110	57	51.81 ± 3.49 ^b^	24.91 ± 2.33 ^a^	25.13 ± 1.62 ^a^	31.42 ± 2.90 ^a^	***
**Total Others**			124.5 ± 4.06	112.2 ± 2.93	121.0 ± 3.65	114.6 ± 4.35	ns
2,3-Butanedione	563	86	14.39 ± 1.09 ^a,b^	17.62 ± 1.09 ^c^	11.79 ± 0.45 ^a^	16.51 ± 0.96 ^b,c^	***
2-Butanone	569	72	32.67 ± 2.16 ^a^	58.90 ± 2.54 ^c^	60.79 ± 1.61 ^c^	45.88 ± 1.52 ^b^	***
3-Pentanone	722	57	18.34 ± 1.30 ^b^	10.49 ± 1.46 ^a^	16.93 ± 1.03 ^b^	16.74 ± 0.95 ^b^	***
Acetoin	785	45	76.61 ± 3.67 ^a^	151.9 ± 6.34 ^c^	132.3 ± 4.82 ^b^	138.8 ± 3.58 ^b,c^	***
2-Heptanone	985	58	3.58 ± 0.94 ^b^	1.27 ± 0.05 ^a^	1.90 ± 0.17 ^a^	6.31 ± 0.25 ^c^	***
Butyrolactone	1072	86	17.11 ± 0.57 ^a^	36.26 ± 1.01 ^b^	41.24 ± 1.51 ^c^	37.04 ± 1.06 ^b^	***
**Total Ketones**			162.7 ± 4.96 ^a^	276.4 ± 9.39 ^b^	265.0 ± 6.57 ^b^	261.2 ± 5.47 ^b^	***
Acetic acid	676	60	1513 ± 40.31 ^a^	2088 ± 69.46 ^b^	2013 ± 44.52 ^b^	1964 ± 58.87 ^b^	***
Butanoic acid	932	60	341.6 ± 13.55 ^a^	578.7 ± 25.65 ^c^	683.7 ± 23.27 ^d^	513.7 ± 14.79 ^b^	***
Butanoic acid, 3-methyl-	986	60	18.87 ± 1.02 ^a^	34.33 ± 1.72 ^b^	37.57 ± 1.33 ^b^	36.75 ± 1.46 ^b^	***
Pentanoic acid	1029	60	13.40 ± 1.15 ^a^	17.94 ± 0.52 ^b^	22.04 ± 0.70 ^c^	20.46 ± 0.97 ^c^	***
Hexanoic acid	1117	60	55.30 ± 2.41 ^a^	65.52 ± 2.51 ^b^	68.80 ± 2.49 ^b,c^	74.99 ± 3.03 ^c^	***
**Total Acids**			1943 ± 46.64 ^a^	2785 ± 85.03 ^b,c^	2825 ± 55.68 ^c^	2610 ± 71.52 ^b^	***
Propanoic acid, ethyl ester	728	57	28.23 ± 1.93 ^a^	36.39 ± 1.83 ^b^	52.58 ± 2.42 ^c^	26.61 ± 1.05 ^a^	***
n-Propyl acetate	736	61	2.96 ± 0.17 ^a^	3.96 ± 0.31 ^b^	5.72 ± 0.26 ^c^	2.37 ± 0.07 ^a^	***
Propanoic acid, 2-methyl-, ethyl ester	800	71	4.74 ± 0.31 ^a^	6.60 ± 0.35 ^b^	7.01 ± 0.39 ^b^	4.81 ± 0.18 ^a^	***
Butanoic acid, ethyl ester	860	88	126.8 ± 7.95 ^a^	178.3 ± 8.66 ^c^	198.8 ± 9.69 ^c^	155.2 ± 4.68 ^b^	***
Butanoic acid, 3-methyl-, ethyl ester	925	88	12.87 ± 0.54 ^a^	20.66 ± 1.18 ^b^	21.67 ± 1.21 ^b^	20.41 ± 0.62 ^b^	***
Pentanoic acid, ethyl ester	978	88	10.15 ± 0.93 ^a^	11.67 ± 0.96 ^a^	15.01 ± 1.73 ^b^	11.40 ± 0.62 ^a^	*
Octanoic acid, ethyl ester	1250	88	17.66 ± 0.81 ^a^	19.99 ± 0.72 ^b^	22.12 ± 0.75 ^b^	20.09 ± 0.72 ^b^	**
Decanoic acid, ethyl ester	1397	88	7.17 ± 0.34 ^d^	4.92 ± 0.17 ^b^	5.63 ± 0.18 ^c^	3.82 ± 0.16 ^a^	***
**Total Esters**			210.6 ± 9.73 ^a^	282.5 ± 11.66 ^c^	328.5 ± 11.68 ^d^	244.8 ± 6.75 ^b^	***
Benzene, 1,3-dimethyl-	939	91	5.13 ± 0.19 ^a^	5.51 ± 0.09 ^a,b^	5.34 ± 0.16 ^a,b^	5.67 ± 0.09 ^b^	*
α-Thujene	990	92	380.1 ± 14.23 ^a^	622.9 ± 30.47 ^b^	596.3 ± 31.77 ^b^	579.3 ± 26.52 ^b^	***
1R-α-Pinene	998	93	1208 ± 53.60 ^a^	1745 ± 58.31 ^b^	1728 ± 72.11 ^b^	1688 ± 74.63 ^b^	***
Camphene	1018	93	42.92 ± 3.36 ^a^	57.17 ± 3.08 ^b^	53.59 ± 2.65 ^b^	49.58 ± 2.28 ^a,b^	**
(+)-Camphene	1018	121	41.92 ± 3.27 ^a^	54.49 ± 2.88 ^b^	51.71 ± 2.42 ^b^	47.52 ± 2.51 ^a,b^	*
β-Thujene	1046	136	398.5 ± 17.13 ^a^	658.7 ± 28.11 ^b^	634.3 ± 29.62 ^b^	605.2 ± 29.37 ^b^	***
Pseudolimonene	1048	121	192.0 ± 10.53 ^a^	233.9 ± 11.56 ^b^	241.9 ± 13.38 ^b^	229.7 ± 14.21 ^b^	*
β-Myrcene	1058	93	380.4 ± 20.94 ^a^	692.0 ± 23.94 ^b^	725.1 ± 28.23 ^b^	692.2 ± 31.09 ^b^	***
α-Phellandrene	1075	93	471.7 ± 27.49 ^a^	980.3 ± 34.30 ^b^	1025 ± 57.47 ^b^	934.0 ± 41.24 ^b^	***
3-Carene	1078	121	373.6 ± 23.83 ^a^	685.0 ± 30.62 ^b^	661.0 ± 31.60 ^b^	712.9 ± 36.69 ^b^	***
α-Terpinene	1087	121	236.4 ± 20.23 ^a^	416.7 ± 17.56 ^b^	465.2 ± 15.54 ^c^	397.7 ± 14.10 ^b^	***
D-Limonene	1098	79	460.9 ± 8.99 ^a^	707.0 ± 18.02 ^b^	770.7 ± 27.49 ^c^	682.5 ± 22.11 ^b^	***
o-Cymene	1102	119	2074 ± 78.00 ^a^	2702 ± 103.42 ^c^	2657 ± 85.9 ^b,c^	2426 ± 95.25 ^b^	***
β-Phellandrene	1103	93	940.3 ± 57.34 ^a^	1418 ± 56.98 ^b^	1592 ± 58.51 ^c^	1484 ± 53.9 ^b,c^	***
Eucalyptol	1109	154	12.35 ± 0.33 ^a^	14.70 ± 0.20 ^b^	16.13 ± 0.35 ^c^	14.56 ± 0.25 ^b^	***
β-Ocimene	1114	91	5.76 ± 0.50 ^a^	9.41 ± 0.86 ^b^	12.11 ± 0.53 ^c^	10.42 ± 0.47 ^b,c^	***
δ-Terpinene	1125	77	147.0 ± 9.96 ^a^	228.4 ± 11.26 ^b^	233.5 ± 14.97 ^b^	229.8 ± 7.73 ^b^	***
(+)-4-Carene	1152	121	137.1 ± 9.85 ^a^	237.7 ± 12.49 ^b^	261.5 ± 16.38 ^b^	246.0 ± 9.91 ^b^	***
Benzyl alcohol	1162	79	66.16 ± 2.94 ^a^	78.58 ± 2.00 ^b^	102.1 ± 3.00 ^d^	90.14 ± 3.31 ^c^	***
m-Cymenene	1167	117	98.41 ± 5.89 ^a^	124.1 ± 7.87 ^b^	117.5 ± 7.13 ^b^	97.66 ± 4.68 ^a^	**
trans-4-Thujanol	1196	71	16.67 ± 0.60 ^a^	27.56 ± 0.84 ^b^	32.04 ± 1.14 ^c^	27.23 ± 1.02 ^b^	***
p-Cresol	1223	107	7.64 ± 0.39 ^a^	11.17 ± 0.39 ^b^	11.71 ± 0.58 ^b^	11.78 ± 0.49 ^b^	***
Phenylethyl Alcohol	1226	91	31.21 ± 1.19 ^a^	37.32 ± 2.55 ^b,c^	39.09 ± 1.35 ^c^	32.83 ± 1.39 ^a,b^	**
α-Phellandren-8-ol	1251	91	31.67 ± 1.40 ^a^	38.76 ± 0.98 ^b,c^	41.97 ± 0.95 ^c^	37.12 ± 1.35 ^b^	***
Terpinen-4-ol	1254	71	187.4 ± 6.69 ^a^	253.4 ± 7.80 ^b^	291.8 ± 6.59 ^c^	250.2 ± 9.24 ^b^	***
α-Terpineol	1271	121	9.73 ± 0.35 ^a^	15.19 ± 0.43 ^b^	16.90 ± 0.42 ^c^	14.54 ± 0.61 ^b^	***
Safrole	1340	162	430.0 ± 14.41 ^a^	679.0 ± 16.97 ^c^	712.3 ± 13.91 ^c^	624.4 ± 23.68 ^b^	***
δ-Elemene	1357	121	25.12 ± 0.90 ^a^	57.76 ± 2.18 ^b,c^	62.67 ± 2.92 ^c^	54.74 ± 3.48 ^b^	***
α-Cubebene	1365	161	9.03 ± 0.55 ^a^	14.77 ± 0.50 ^b^	17.54 ± 0.65 ^c^	15.52 ± 0.83 ^b^	***
Copaene	1388	161	93.26 ± 5.16 ^a^	147.7 ± 5.24 ^b^	174.6 ± 5.65 ^c^	155.7 ± 7.62 ^b^	***
Methyleugenol	1422	178	12.88 ± 0.42 ^a^	23.53 ± 0.60 ^c^	25.74 ± 1.01 ^d^	21.35 ± 0.82 ^b^	***
Caryophyllene	1430	133	258.8 ± 9.19 ^a^	451.8 ± 16.22 ^b^	481.3 ± 20.21 ^b^	455.6 ± 21.50 ^b^	***
Myristicin	1493	192	31.03 ± 0.99 ^a^	51.40 ± 1.23 ^b^	56.88 ± 1.07 ^c^	49.53 ± 1.69 ^b^	***
Elemicin	1502	208	3.94 ± 0.13 ^a^	7.17 ± 0.14 ^c^	6.87 ± 0.13 ^c^	5.92 ± 0.17 ^b^	***
**Total Terpenoids and Benzene-Derive Compounds**			8821 ± 303.2 ^a^	13,490 ± 334.8 ^b^	13,923 ± 407.1 ^b^	12,981 ± 388.7 ^b^	***
**TOTAL COMPOUNDS**			12,378 ± 324.4 ^a^	18,441 ± 373.7 ^b,c^	19,027 ± 446.6 ^c^	17,854 ± 369.8 ^b^	***

^a–d^ Mean values in the same row (corresponding to the same parameter) not followed by a common letter differ significantly (*p* < 0.05; Duncan test); Sig.: significance: * (*p* < 0.05), ** (*p* < 0.01), *** (*p* < 0.001), ns (not significant); SEM: standard error of the mean; LRI: linear retention index calculated for DB-624 capillary column (J&W scientific: 30 m × 0.25 mm id, 1.4 µm film thickness) installed on a gas chromatograph equipped with a mass selective detector; *m*/*z*: quantifier ion. Treatments: CON: sausages prepared 100% pork fat; OLI: sausages reformulated with 50% of pork fat replaced by olive oil; CAN: sausages reformulated with 50% of pork fat replaced by canola oil; SOY: sausages reformulated with 50% of pork fat replaced by soy oil.
